# Staged osteotomy in lateral position for the treatment of severe kyphotic deformity secondary to ankylosing spondylitis: a retrospective study

**DOI:** 10.1186/s13018-023-03884-5

**Published:** 2023-06-09

**Authors:** Zhengjun Hu, Rui Zhong, Deng Zhao, Fei Wang, Huaqiang Huang, Dengxu Jiang, Zhong Zhang, Yijian Liang

**Affiliations:** Department of Spine Surgery, The Third People’s Hospital of Chengdu, Southwest Jiaotong University, #82 Qinglong Street, Chengdu, 610031 Sichuan China

**Keywords:** Ankylosing spondylitis, Kyphosis, Staged surgery, Osteotomy, Lateral decubitus position

## Abstract

**Background:**

Patients with severe kyphotic deformity (Cobb > 100°) secondary to ankylosing spondylitis (AS) occasionally cannot undergo corrective surgery in the prone position. Osteotomy in the lateral position might provide a possible solution. In this study, we aim to evaluate the clinical efficacy and safety of staged osteotomy in the lateral position for the treatment of AS-related severe kyphosis with a minimum of 2-year follow-up.

**Methods:**

In total, 23 patients who underwent staged osteotomy in the lateral position from October 2015 to June 2017 were analyzed. In the first stage of surgery, all but one patient underwent a single-level Ponte osteotomy, which was followed by a pedicle subtraction osteotomy in the second stage. Mean follow-up was 30.8 ± 4.6 months. Global kyphosis (GK), thoracic kyphosis (TK), lumbar lordosis (LL), sagittal vertical axis (SVA), osteotomized vertebra intervertebral angle (OVI), chin-brow vertical angle (CBVA), Oswestry Disability Index (ODI) score and Scoliosis Research Society-22 Patient Questionnaire (SRS-22) were all compared pre- and postoperation.

**Results:**

All kyphosis parameters were significantly improved (all *P* < 0.05). GK was corrected from 115.0 ± 13.4° to 46.5 ± 9.0° postoperatively, with a mean correction of 68.5°. SVA was improved from 21.2 ± 5.1 cm to 5.1 ± 1.8 cm postoperatively. After surgery, CBVA was adjusted from 64.1 ± 23.2° to 5.7 ± 10.6° and OVI was changed from 9.0 ± 2.7° to − 20.1 ± 5.6°. Both the ODI and SRS-22 showed substantial improvements (all *P* < 0.05). Four patients with mild complications were observed perioperatively.

**Conclusion:**

In AS patients with severe kyphosis, satisfactory correction can be safely achieved with staged osteotomy in the lateral position, which can not only correct the sagittal imbalance of the spine with acceptable complications but also facilitate the placement of the intraoperative position.

## Background

Ankylosing spondylitis (AS) is a chronic non-specific inflammatory disease involving fibro-osseous tissues[1, 2]. Advanced AS patients often suffer from severe rigid thoracolumbar kyphosis (Cobb > 100°), resulting in sagittal-plane imbalance, atypical appearance and limited horizontal visual fields. In more severe cases, cardiopulmonary and digestive functions may also be impaired due to the compression of thoracic and abdominal viscera [3–6]. Corrective osteotomy is the only effective treatment for AS patients with kyphosis. A variety of osteotomies have been reported for kyphosis correction, including Smith-Peterson osteotomy (SPO), Ponte osteotomy (PO), pedicle subtraction osteotomy (PSO) and vertebral column resection (VCR) [7–9]. SPO is a posterior chevron-shaped osteotomy that obtains 10° correction with a single level [7, 10]. Ponte osteotomy and SPO are often mistakenly used in scientific literature. The key distinctions between these two osteotomies lie in Schwab's Osteotomy Classification and the extent of resection. Ponte osteotomy necessitates adequate laminae resection to rectify thoracolumbar kyphosis by significantly shortening the posterior column [11]. PSO is a closing wedge osteotomy that does not lengthen the anterior column and accomplishes a correction of approximately 30° to 40°. The aforementioned osteotomies are all based on the prone position; however, positioning AS patients with severe kyphosis in the prone position can be challenging, even with the aid of a specially designed folding bed. One-stage two-level PSO has been reported as an effective treatment for these patients. Nonetheless, this procedure is technically demanding for surgeons and anesthetists, potentially resulting in more intraoperative bleeding and a higher incidence of neurological complications [12–14]. In order to reduce the technical complexity of correcting severe kyphosis in AS patients and to address the issue of intraoperative position setting, staged osteotomy in the lateral position was initially proposed for the treatment of severe kyphotic deformity secondary to ankylosing spondylitis.

Therefore, the aim of this study was to evaluate the clinical efficacy and safety of staged osteotomy in the lateral position for the treatment of AS-related severe kyphosis with a minimum of 2-year follow-up.

## Methods

### Patients

A retrospective analysis was conducted on AS patients with severe kyphotic deformity who underwent staged osteotomy in the lateral position from October 2015 to June 2017. AS was diagnosed according to the modified New York standards proposed in 1984 [15]. Indications for corrective surgery encompassed severe kyphotic appearance, difficulty in forward gazing, limitations in daily living and impairment of cardiopulmonary function. Inclusion criteria included: (1) AS patient with a Cobb angle of thoracolumbar kyphosis > 100°; (2) staged osteotomy performed in the lateral position; (3) a minimum follow-up of 2 years with complete clinical data. Exclusion criteria were: (1) previous history of spinal surgery; (2) severe concomitant diseases, such as spinal tumors or infections. The study was approved by the Ethics Committee of the Third People's Hospital of Chengdu.

The study involved 23 patients (20 males and 3 females) with a mean age of 43.5 ± 9.5 years (range 29–67 years). The follow-up endpoint was in November 2019, with an average follow-up time of 30.8 ± 4.6 months (range 25–40).

No patients exhibited neurological deficits prior to surgery. According to the 2005 ATS/ERS pulmonary function classification standard [16], all but one patient demonstrated poor lung function. Table 1 summarizes the findings of the patient characteristics in this study.

**Table 1:** Patient characteristics

Case	Gender	Age(y)	Apical	Pseudarthrosis	Pulmonary function	Hip function	Follow-up(m)
1	M	42	T12/L1	T12/L1	Severe	Bilateral NR	40
2	F	41	T12	–	Moderate	Right NR	25
3	M	43	T8/9	–	Moderately severe	Left AK	25
4	M	41	L1	–	Very severe	Bilateral NR	33
5	F	67	T12	T12/L1	Very severe	Right NR	28
6	M	33	T12	–	Moderate	Left AK	26
7	M	37	T12/L1	–	Very severe	Bilateral AK	26
8	M	35	T10	T11/T12	Mild	Bilateral AK	32
9	M	48	L1	L3/4	Moderate	Right NR	32
10	M	33	T12/L1	–	Moderate	Right NR	36
11	M	51	T10/T11	–	Moderate	Bilateral NR	32
12	M	44	T12	T12/L1	Moderate	Bilateral NR	31
13	M	40	L2	–	Moderate	Bilateral NR	40
14	M	29	T11	–	Moderate	Normal	29
15	M	39	T12	–	Mild	Bilateral NR	30
16	M	55	T12	–	Severe	Bilateral AK	26
17	F	58	T11/12	–	Mild	Bilateral NR	32
18	M	40	T11/12	–	Normal	Bilateral NR	34
19	M	37	T10/T11	–	Moderate	Bilateral NR	38
20	M	45	T12	–	Severe	Right NR	25
21	M	33	T12	–	Moderately severe	Left AK	31
22	M	56	T12	–	Mild	Bilateral NR	27
23	M	54	T12	–	Moderate	Left NR	30

*NR* significantly narrowing of hip joint, *AK* ankylosing of hip joint

### Radiographic and clinical assessment

Radiologic and clinical parameter data were collected and evaluated for patients preoperatively, postoperatively and at last follow-up. A senior spine surgeon, not involved in the surgery, measured these parameters. Each parameter was measured three times and averaged. All radiographic measurements were taken on standing full-length spine lateral radiographs by an independent spine surgeon, which included: (1) global kyphosis (GK): defined as the angle between the maximally tilted upper and lower endplate of the vertebral body; (2) thoracic kyphosis (TK): the angle between the superior endplate of T5 and the inferior endplate of T12; (3) lumbar lordosis (LL): the angle between the upper endplate of T12 and S1; a positive value for the aforementioned angle indicated kyphosis; (4) sagittal vertical axis (SVA): the distance between the C7 plumb line and the posterosuperior corner of S1, which is positive if the C7 plumb line was anterior to the posterosuperior corner of S1 [17].

**Fig. 1:**
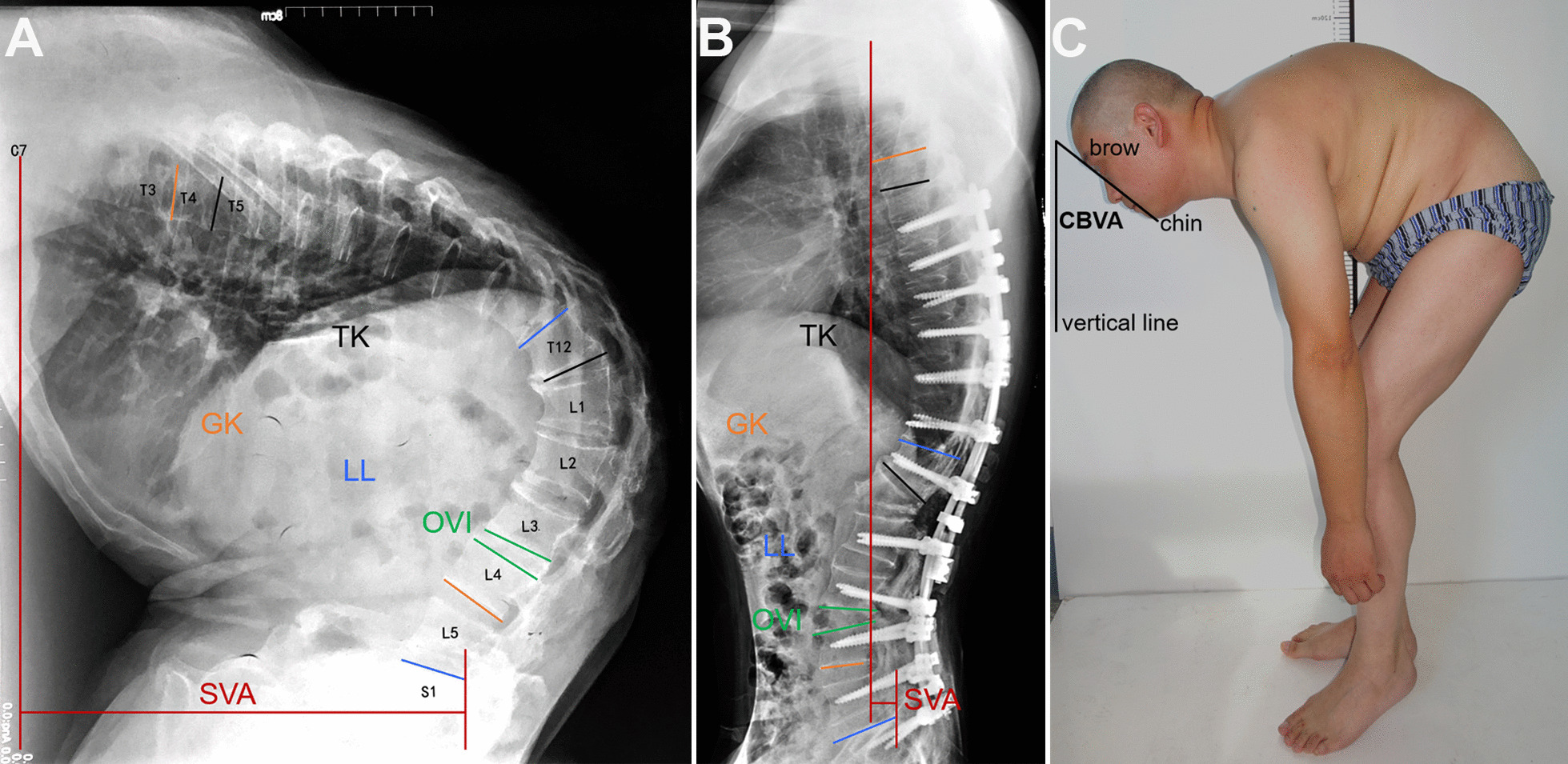
Pre- and postoperative radiographic parameter measurements diagrams (**A,** **B**)

During the first stage, 22 patients underwent a single-level Ponte osteotomy in the lateral position. The correction result of the first-stage surgery was assessed by the osteotomized vertebra intervertebral angle (OVI), with positive values indicating kyphosis (Fig. 1A, B).

Chin-brow vertical angle (CBVA) was measured using standing lateral photographs of patients, defined as the angle between a line from the chin to the brow and its plumb line [18].

Clinical efficacy assessments included the Oswestry Disability Index (ODI) score and the Scoliosis Research Society-22 Patient Questionnaire (SRS-22) [19, 20].

### Surgical technique

**Fig. 2:**
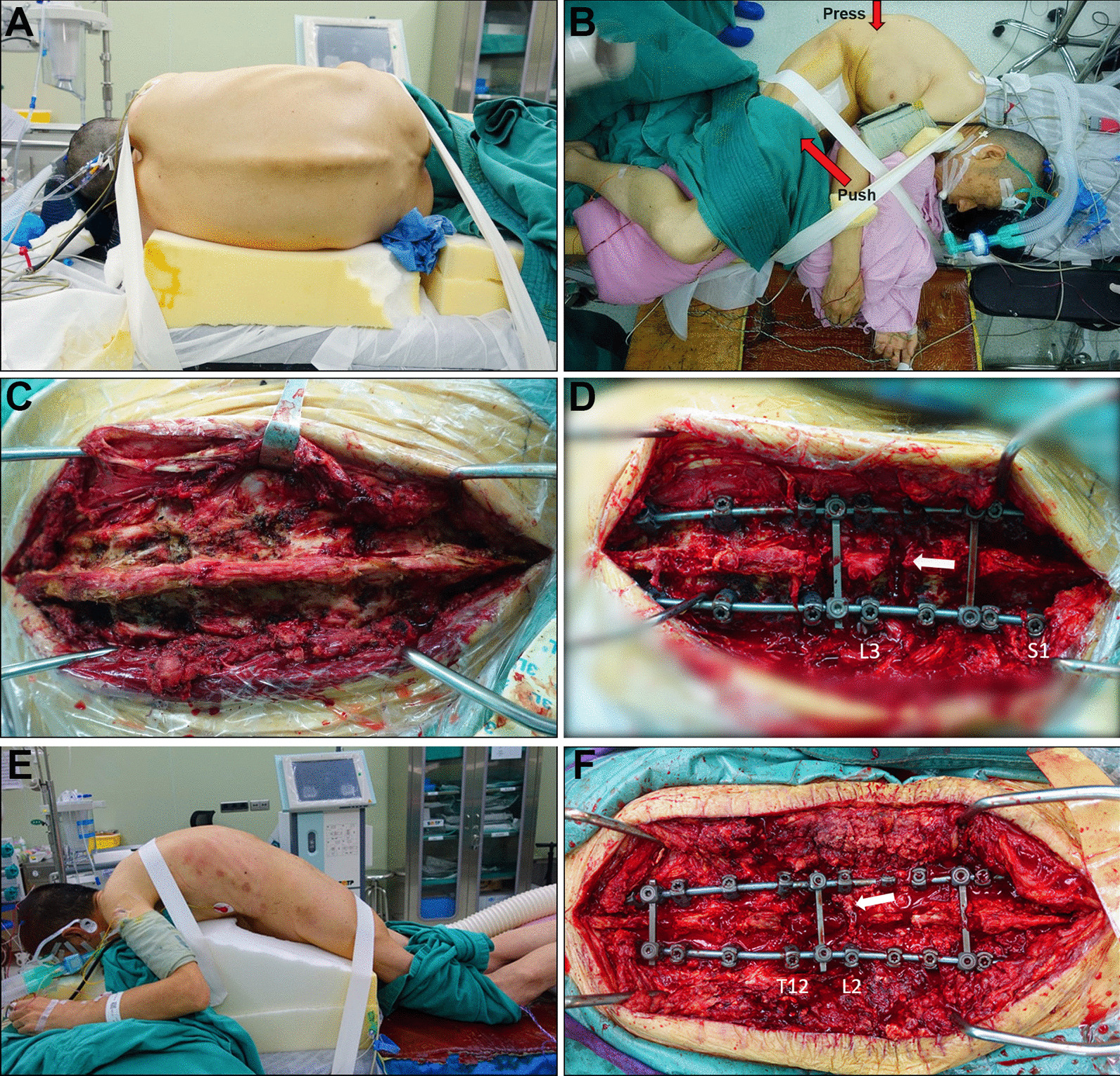
Lateral position placement (**A**, **B**). First-stage L3/4 Ponte osteotomy in lateral position pre- and postosteotomy procedure (**C**, **D**). Second-stage L1 PSO in prone position (**E**, **F**). Red arrows demonstrate the correction process accomplished by slowly pushing the front of the thigh backward and pressing the osteotomy site simultaneously. White arrows indicate the closed osteotomy gap

**Fig. 3:**
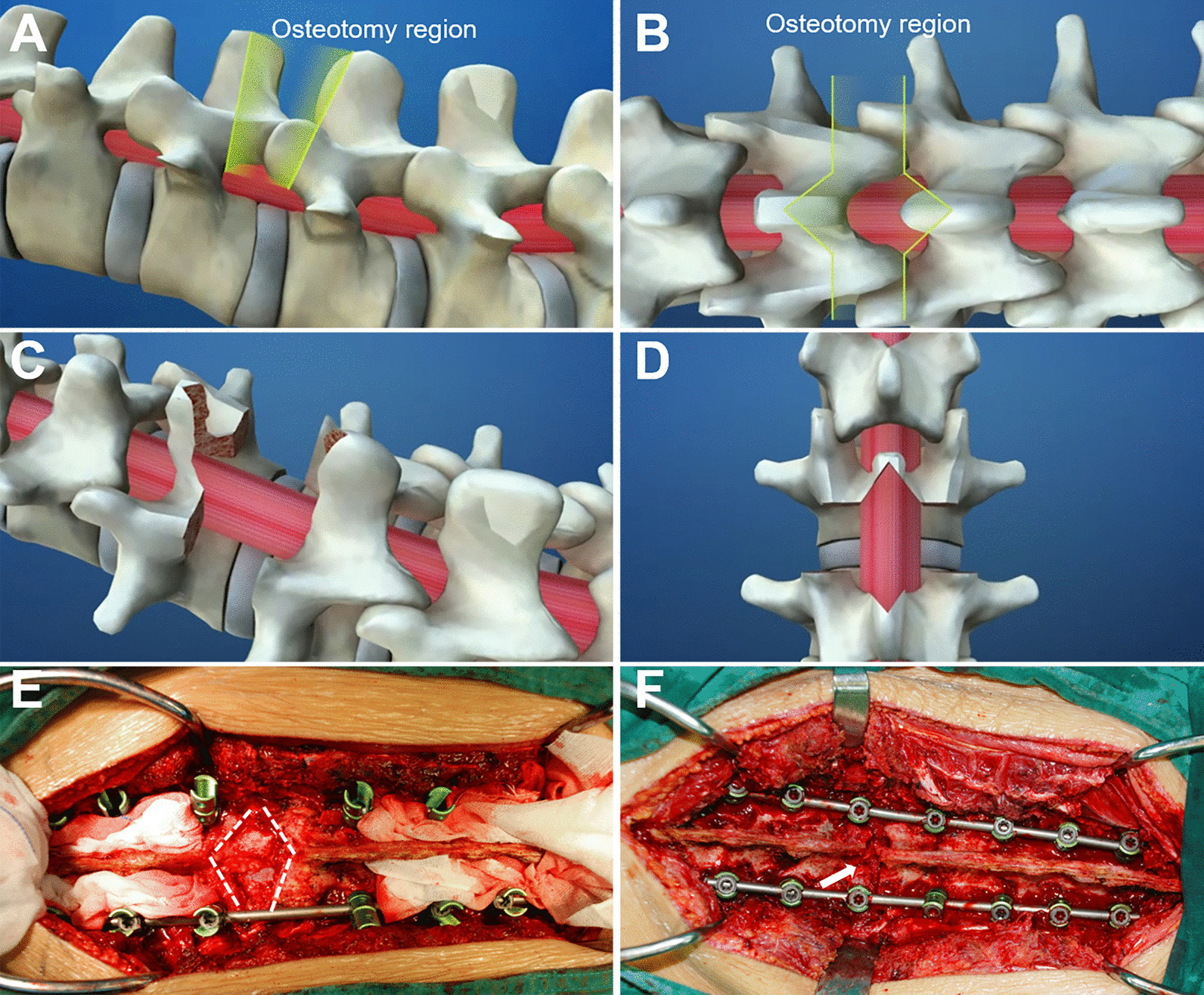
Illustration of Ponte osteotomy from pedicle to pedicle. The ideal osteotomy region (**A**–**D**). A unilateral temporary rod was installed after screw instrumentation; white dotted lines display the actual region of Ponte osteotomy, approximately a quadrangle shape (**E**). White arrows indicate the closed osteotomy gap after PO (**F**)

(1) In the first stage of lateral osteotomy, all patients underwent a single-level Ponte osteotomy, with the exception of one patient who required L1 PSO due to extremely severe kyphosis. Following general anesthesia, patients were positioned laterally on a customized foam pad that offered ample support for the head, thorax, hip and lateral lower extremity and subsequently secured with medical tape (Fig. 2A, B). The posterior components were exposed as far as the transverse process (Fig. 2C). Bilateral pedicle screws were implanted. As the screws were inserted in the lateral position, the inherent habit of screw instrumentation in the prone position needed to be altered. A unilateral temporary rod was placed to prevent uncontrolled sagittal translation during the osteotomy. The Ponte osteotomy was then performed from pedicle to pedicle [11]. The correction was achieved by gradually closing the osteotomy gap through slowly pushing back on the anterior thigh and simultaneously applying pressure on the osteotomy site (Fig. 3).

(2) The second-stage PSO, based on Wang's method [21], was conducted two weeks after the first-stage surgery. All patients, except one, underwent the second-stage procedure in the prone position. The corresponding author performed all surgeries under multimodal neuroelectrophysiology monitoring (Fig. 2E, F).

### Statistical analysis

Statistical analysis was carried out using SPSS 18.0 (SPSS Inc, Chicago, IL, USA). All measurements were reported as mean ± standard deviation (SD). One-way ANOVA was utilized to compare data for GK, TK, LL, OVA, SVA, CBVA, ODI and SRS-22 scores preoperatively, postoperatively, and at the last follow-up. Statistical significance was considered at *P* < 0.05.

## Results

### Surgical results

All patients exhibited significant improvement in stooped and downward-looking posture after surgery. Table 2 summarizes the surgical characteristics findings. The average operation interval between the primary and secondary surgery was (23.5 ± 6.8) days.

**Table 2:** Surgical characteristics

Case	First stage			OP Interval (d)	Second stage			
	OT(m)	BL (mL)	PO Level		OT (m)	BL (ml)	PSO Level	Fusion Level
1	275	900	L3-4	22	222	1100	L1	T9-S1
2	200	550	L3-4	24	236	950	L1	T9-L5
3	165	500	L3-4	19	210	2100	T8	T4-L5
4	225	1200	L3-4	23	195	800	L1	T9-L5
5	170	500	L3-4	23	225	900	L1	T9-L5
6	150	600	L3-4	26	310	1000	T12	T8-L5
7	320	1600	N	42	375	2600	L4	T9-S1
8	175	800	L3-4	40	205	900	T12	T8-S1
9	230	500	L3-4	16	270	1000	L1	T9-L5
10	200	1000	L3-4	33	232	1500	L1	T6-S1
11	255	700	L3-4	21	285	1300	L1	T9-L5
12	235	1100	L2-3	21	290	1400	T12	T8-L5
13	200	650	L2-3	19	270	1000	L1	T9-L5
14	210	1200	L3-4	21	200	1200	L1	T9-S1
15	255	800	L3-4	19	255	1200	L1	T9-S1
16	190	700	L3-4	20	272	1000	L1	T9-S1
17	205	800	L2-3	22	170	1300	T12	T8-L5
18	210	1000	L3-4	21	230	1100	L1	T9-S1
19	150	400	L3-4	21	265	1500	T12	T8-S1
20	210	750	L3-4	22	243	1100	L1	T9-S1
21	245	950	L1-2	30	230	900	T12	T8-S1
22	175	400	L2-3	22	215	600	L1	T8-L5
23	230	1000	L2-3	14	205	900	L1	T9-L5

*OT* operation time (min), *BL* blood loss (mL), *OP* operation, *PSO* pedicle subtraction osteotomy, *PO* Ponte osteotomy

N indicates not applicable, this patient underwent L1 PSO at first stage of surgery

In the first stage of surgery, 16 patients (72.7%) underwent L3-4 Ponte osteotomy (Table 2). The mean operative time was (207 ± 34) minutes, and blood loss was (773 ± 247) mL (Table 3).

**Table 3:** Intraoperative parameters

Parameters	First stage	Second stage	Total
Operation time, min	212 ± 41	244 ± 45	456 ± 71
Blood loss, mL	809 ± 297	1189 ± 434	1998 ± 613

In the second-stage PSO procedure, the average surgical time was (244 ± 45) minutes, and blood loss was (1189 ± 434) mL (Table 3); PSO level: 15 cases (68.2%) L1. Four patients with pseudarthrosis underwent PSO through pseudarthrosis. One case with L3/4 level pseudarthrosis underwent PSO above the pseudarthrosis. Final fusion: T9-L5 and T9-S1 were the most common fusion segments, with 7 cases each. In this study, the instrumentation range typically encompassed 4 levels above and 3 levels below the osteotomy vertebra (56.5%).

### Radiologic and chin brow–vertical angle results

After surgery, all kyphosis parameters significantly improved, and the sagittal balance of the spine was substantially restored (all *P* < 0.05). No patient showed loss of correction at the last follow-up (all *P* > 0.05) (Table 4). The preoperative GK was (115.0 ± 13.4)°, which was corrected to (46.5 ± 9.0)° postoperatively and (47.5 ± 8.8)° at the last follow-up, with a mean correction of 68.5° and 67.5°, respectively. The mean SVA improved from (21.2 ± 5.1) cm preoperatively to (5.1 ± 1.8) cm postoperatively, with a mean correction of 16.1 cm.

**Table 4:** The comparison of sagittal parameters of spine preoperatively, postoperatively, and at the latest follow-up

Parameters	Pre-op	Post-op	Final	*P*
GK (°)	115.0 ± 13.4	46.5 ± 9.0	47.5 ± 8.8	< 0.001
TK (°)	60.2 ± 18.9	43.7 ± 11.8	44.9 ± 13.4	0.001
LL (°)	40.7 ± 24.3	− 41.8 ± 13.4	− 41.4 ± 13.2	< 0.001
SVA (cm)	21.2 ± 5.1	5.1 ± 1.8	5.0 ± 2.0	< 0.001
CBVA (°)	64.1 ± 23.2	5.7 ± 10.6	6.3 ± 10.7	< 0.001
OVI(°)	9.0 ± 2.7	− 20.1 ± 5.6	− 19.6 ± 5.7	< 0.001

*GK* global kyphosis, *TK* thoracic kyphosis, *LL* lumbar lordosis, *SVA* sagittal vertical axis, *CBVA* chin-brow vertical angle, *OVA* osteotomized vertebra intervertebral angle, *P* Post-op or final versus Pre-op

The OVI was (9.0 ± 2.7)° preoperatively, significantly corrected to − (20.1 ± 5.6)° postoperatively and − (19.6 ± 5.7)° at the last follow-up (*P* < 0.01), with a mean correction of 29.1° and 28.6°, respectively.

The mean CBVA of the patients before surgery was (64.1 ± 23.2)°, which significantly improved to (5.7 ± 10.6)°postoperatively and (6.3 ± 10.7)° at the last follow-up (*P* < 0.01).

**Fig. 4:**
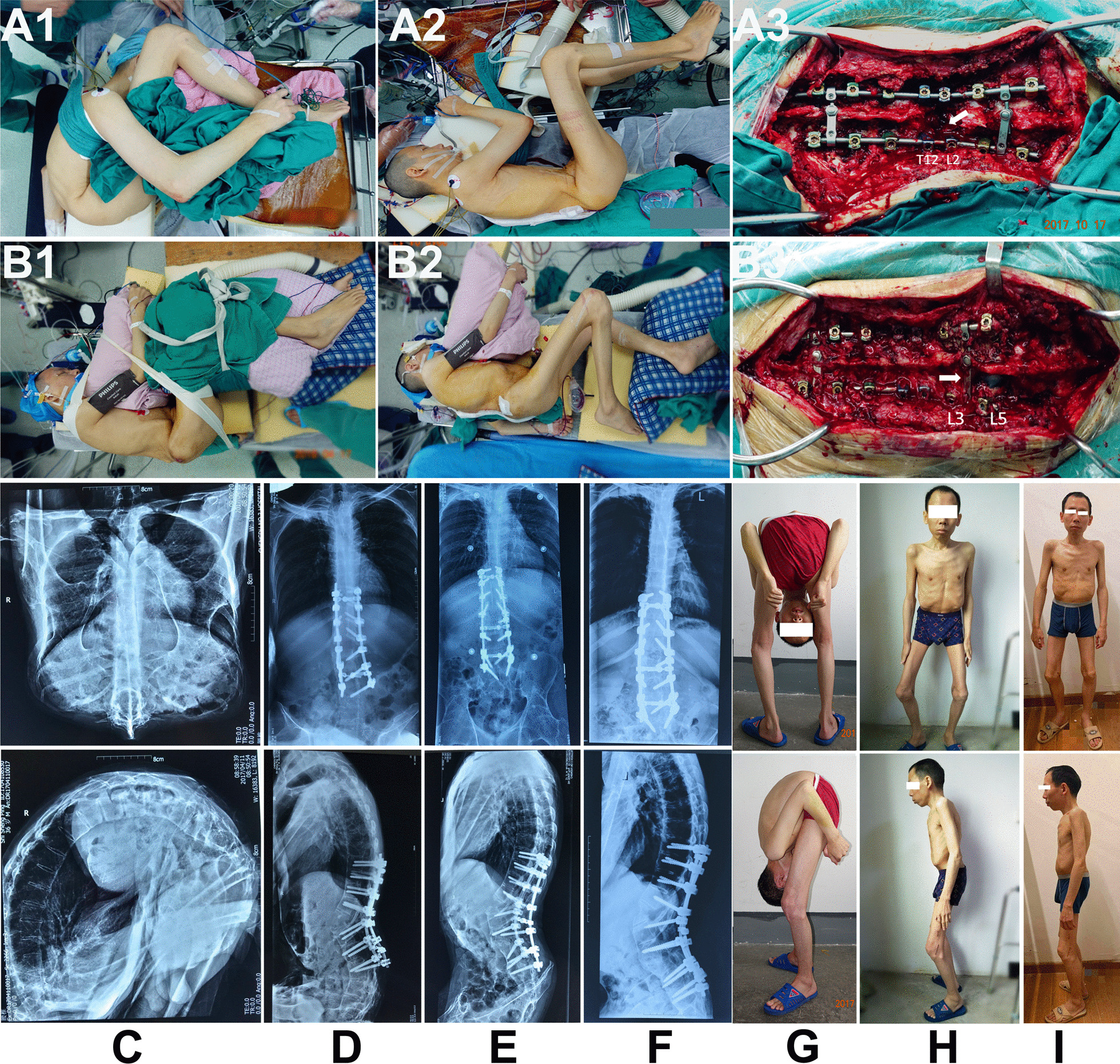
A 35-year-old man with a completely folded spine, who cannot undergo corrective surgery in a prone position. **A1**–**A3** display the first-stage L1 PSO in lateral position; **B1**–**B3** exhibit the second-stage L4 PSO in lateral position. **C**–**F** present preoperative, post-first-stage osteotomy, post-second-stage osteotomy and final follow-up radiographs. **G**–**I** depict preoperative, immediate postoperative and 31 months postoperative appearances. White arrows indicate osteotomy level

Figure 4 presents a typical case of a 35-year-old "folded patient" with extremely severe kyphosis and hip ankylosis who underwent L1 and L4 PSO in two stages and the final total hip arthroplasty. The GK was corrected from 158° to 62°.

### Clinical assessment

The mean ODI score of the patients before surgery was 34.7 ± 10.1, which significantly decreased to 15.6 ± 6.6 postoperatively and 15.2 ± 7.0 points at the last follow-up (*P* < 0.01) (Table 5). After surgery, all patients experienced a significant improvement in quality of life; the four components of the SRS-22 significantly improved after surgery (all *P* < 0.05; Table 5).

**Table 5:** The comparison of SRS-22 and ODI score preoperatively, postoperatively, and at the latest follow-up

Variables	Pre-op	Post-op	Final	*P*
Pain	13.7 ± 3.1	21.8 ± 2.4	22.6 ± 1.8	< 0.001
Mental health	14.5 ± 3.8	21.4 ± 1.9	22.0 ± 2.1	< 0.001
Self-image	9.8 ± 3.7	19.5 ± 3.0	20.3 ± 2.2	< 0.001
Function/activity	15.7 ± 4.2	19.4 ± 3.0	19.5 ± 3.1	0.001
Satisfaction with surgery	N	8.9 ± 1.1	9.4 ± 1.0	N
ODI	34.7 ± 10.1	15.6 ± 6.6	15.2 ± 7.0	< 0.001

*N* not applicable, *P* post-op or final versus pre-op

### Complications

No neurological deficits, vascular injuries or other severe complications were documented. Furthermore, at the minimum 2-year follow-up, all patients exhibited no occurrence of proximal junctional kyphosis (PJK), distal junctional kyphosis (DJK) or internal fixation-related complications. Additionally, fusion of the osteotomy was achieved in all patients at the most recent follow-up. In total, four patients with complications were identified among 46 operations in 23 patients (8.7%). In the first stage of surgery, one patient developed a superficial infection that was resolved by increasing the frequency of dressing changes, resulting in a complication incidence of 4.3% (1/23). In the second-stage surgery, three patients experienced postoperative cerebrospinal fluid leakage, which was addressed by prolonging the drainage time with a pressure dressing; the complication incidence for the second-stage surgery was 13.0% (3/23).

## Discussion

SPO, PSO, Ponte and VCR are the four corrective osteotomies reported so far for the treatment of AS-related kyphosis, all of which are based on the prone position. However, positioning AS patients with severe kyphosis in the prone position can be challenging, often requiring a specially prepared reverse "V"-shaped folding bed. In some cases, patients cannot undergo corrective surgery in the prone position due to the attachment of the chest and abdomen (Fig. 4). Corrective surgery in the lateral position provides an alternative approach to overcome the difficulty of intraoperative position setting. In this study, only a simple foam pad was required to complete the lateral position without using any special equipment, which would reduce the preoperative preparation time. Regardless of the severity of kyphosis, or even if the patient is "folded," achieving the lateral position remains convenient. During the correction process in the lateral position, an off-table assistant can observe the correction effect more directly from the ventral perspective, aiding in the assessment of whether the correction is excessive or insufficient. Blindness, a frequent consequence during prone spine surgery, can be caused by local extrusion and ischemic optic neuropathy [22]. Qian et al. [23] reported that brachial plexus palsy may occur during prone spinal osteotomy due to local compression and excessive abduction of the shoulder. The absence of local compression in the lateral position allows for the avoidance of postoperative blindness and brachial plexus paralysis.

One-stage two-level PSO is regarded as an effective treatment for severe AS-related kyphosis since it can correct an angle of 100°. However, this method is technically challenging, necessitating a longer operation time and more blood loss, which increases the difficulty and risk of surgery [24, 25]. Zhong et al. [26] reported a mean blood loss of 2560 ± 1109 ml in 10 AS patients with severe kyphosis who were treated with one-stage two-level PSO. Zhang et al. [27] also reported that the mean blood loss was 3311 ± 523 ml in 9 patients with severe kyphotic AS who received a one-stage operation. In this study, the total blood loss in staged osteotomies was 1998 ± 613 ml, which was less than that of the one-stage two-level PSO. Compared to PSO, the Ponte osteotomy performed in the first-stage surgery is less aggressive, results in less blood loss and carries a lower surgical risk. In this study, all patients with severe kyphosis (Cobb angle > 100°) typically cannot tolerate one-stage two-level osteotomies due to poor physical condition and chronic nutritional deficiencies, which adversely affect coagulation and bleeding [28]. With less blood loss, the older age of the patients enrolled in this study was safer during perioperative period. Consequently, staged surgery is believed to be a relatively safe method for treating severe kyphosis in AS patients, reducing the complexity and risk of surgeries.

In this study, GK, TK, LL, SVA and CBVA were utilized to evaluate the sagittal balance of the spine. Our results demonstrated significant improvements in all parameters after surgery and at the last follow-up. Qian et al. [29] investigated the feasibility of using a single-stage skipping two-level PSO in 10 patients with AS-related severe kyphosis (Cobb > 100°). Their results showed that GK, LL and SVA were corrected from 113.4°, 41.9° and 25.2 cm preoperatively to 71.6°, − 44.1° and 5.8 cm postoperatively. Zhong et al. [26] reported improvements in the kyphosis angle, CBVA and SVA from 92.0°, 37.6° and 24.1 cm preoperatively to 30.0°, − 0.6° and 7.5 cm postoperatively. In the current study, the kyphosis angle was corrected from 115.0° to 46.5° postoperatively, with a mean correction of 68.5°, which was similar to previous studies. Although the Ponte osteotomy performed in the lateral surgery was a Grade 2 osteotomy, it achieved a mean correction of 29.1°. The correction angle was almost equal to the PSO procedure. This might be attributed to the extension of the laminae resection range from pedicle to pedicle, providing an adequate osteotomy gap. Correction is primarily achieved by shortening the posterior column and elongating the anterior column. The wider the osteotomy gap, the greater the correction angle. Unfortunately, this procedure has been associated with elongation of the aorta, and as a result, patients with severe kyphosis undergoing Ponte osteotomy may be subject to aortic injury [30]. However, no severe vascular injury was recorded in this study. This might be due to the absence of abdominal aorta calcification on preoperative CT angiography in all cases, allowing the elastic aorta to adapt well to the spine's elongation during the correction procedure. In our experience, the osteotomy width of the Ponte osteotomy is safe within 25 mm. The degree of kyphosis correction was found to be positively correlated with the extent of aortic lengthening, suggesting that aortic injury was more likely to occur with the correction of more severe kyphosis deformities. In a study by Ji et al. [31], 21 patients with AS who underwent closing-opening wedge osteotomy (COWO) showed aortic length safely increased by an average of 2.2 cm and proposed that the ratio between the increase in aortic length and the correction angle is 1:19.1. In the present study, a Ponte osteotomy within a 25 mm range was performed, resulting in a correction of 29.1°. This approach elongated the aorta by approximately 1.5 cm at a ratio of 1:19.1, which is deemed safer than COWO surgery. Moreover, a study demonstrated that the mean lumbar lamina length is 26.6 mm [32], and the resection range in the current study covers almost the entire lamina. Within the 25 mm osteotomy range, sufficient correction effects can be achieved without increasing the risk of vascular injury; thus, there is no need to extend the scope of laminae resection. The mean SVA improved from 21.2 to 5.1 cm postoperatively with an average correction of 16.1 cm, which was superior to previous studies. In the study by Diao et al. [33], patients with AS-related kyphosis were divided into lordotic and kyphotic lumbar sagittal profiles based on the morphology of the lumbar spine. They suggested that greater correction of kyphosis could be achieved through osteotomy at the lower lumbar vertebra. Additionally, the lower the osteotomy level, the longer the correction lever arm, allowing for more restoration of SVA and better improvement of sagittal balance. However, selecting an osteotomy level below L4 may result in inadequate distal instrumentation segments, leading to complications such as fixation failure and lumbosacral discomfort [34]. In this study, all patients had kyphotic lumbar sagittal profiles. Therefore, L3/4 was the most frequently selected osteotomy level (69.6%) during the initial stage of surgery, which may account for the superiority of our results.

In this study, 23 patients underwent a total of 46 operations in two stages. The complication rate of the first stage, second stage and total was 4.3%, 13% and 8.7%, respectively, which was lower than the average complication rate (13.4%) for osteotomy reported by Qian et al. [29]. Cerebrospinal fluid leakage was the most common complication due to adhesive lesions between the dura and the ligamentum flavum, the facet or lamina [35].

### Limitations

This study also has limitations. The sample size was relatively small. Additionally, retrospective studies have several inherent limitations, such as selection bias and data availability. Sagittal alignment parameters of the spine were evaluated in this study (GK, TK, LL, SVA); however, the spinopelvic alignment parameters (e.g., pelvic incidence, pelvic tilt and sacral slope) were not fully assessed due to the low quality of X-ray imaging.

## Conclusion

In AS patients with severe kyphosis (Cobb > 100°), satisfactory correction is safely achieved by staged osteotomy in the lateral position. This approach not only corrects the sagittal imbalance of the spine with acceptable complications but also facilitates the placement of the intraoperative position.

## Data Availability

Data and materials contributing to this article may be provided by sending an e-mail to the first author.

## Abbreviations

AS: Ankylosing spondylitis
GK: The global kyphosis
TK: Thoracic kyphosis
LL: Lumbar lordosis
SVA: Sagittal vertical axis
OVI: The osteotomized vertebra intervertebral angle
CBVA: Chin-brow vertical angle
ODI: The Oswestry Disability Index
SRS-22: Score and the Scoliosis Research Society-22 Patient Questionnaire
SPO: Smith–Peterson osteotomy
PO: Ponte osteotomy
PSO: Pedicle subtraction osteotomy
VCR: Vertebral column resection
COWO: Closing-opening wedge osteotomy
